# Supplementary material for: Content-rich biological network constructed by mining PubMed abstracts
Source: BMC Bioinformatics. 2004 Oct 8;5:147. doi: 10.1186/1471-2105-5-147 (PMC528731; doi:10.1186/1471-2105-5-147)
Supplement: Additional File 2 — The original results of the above study (non-essential files are deleted to keep the file size under the limit set by BMC bioinformatics). [file 1471-2105-5-147-S2.bz2 › chilibotAdditionalFile2/dip10/158ID7685215E560/html/FOS_TBP.html]

 


 **FOS** and **TBP** 
  
Found 30 abstracts in PubMed, retrieved 10.  
 

 What does Google say? 
 PDF only 
| .edu only 

---

**Interactive relationship** (e.g. stimulation, inhibition, etc)

**Neutral relationship**- The topical application of 50 microg cm2 BaP  [ **TBP** ]  to EpiDerm resulted in the accumulation of BaP  [ **TBP** ]  DNA adducts and c  **fos**  and p53 proteins as evidenced by immunohistochemical localization.  Ref: 9920731 Biochem Biophys Res Commun, 1999
- To examine protein protein interactions with cellular proteins at the visna virus promoter, we used an in vitro protein affinity chromatography assay and electrophoretic mobility shift assay, in addition to an in vivo two hybrid assay, to show that the visna virus Tat protein specifically interacts with the cellular transcription factors  **Fos**  and Jun and the basal transcription factor  **TBP**  TATA binding protein  [ **TBP** ] .  Ref: 9847304 J Virol, 1999

**Non-interactive relationship** (e.g. studied together, co-existance, homology, etc.)

- Mutation of either Tyr248, Tyr357, or Tyr462 to phenylalanine reduced transcription from a c  **fos**  promoter relative to wild type BAP  [ **TBP** ]  TFII I in transfected COS 7 cells, consistent with the interpretation that phosphorylation at these sites contributes to transcriptional activation.  Ref: 11373296 J Biol Chem, 2001
- Strikingly, the first modification observed is methylation of histone H3 lysine 4, which correlates with the binding of the SET9 methyltransferase and the assembly of a complex consisting of c Jun, c  **Fos** , TATA binding protein  [ **TBP** ] , and RNA polymerase II.  Ref: 12588998 Mol Cell Biol, 2003
- These includes nuclear factor,  **FOS**  related antigen 1, cytoplasmic dynein light chain, replication factor C, nucleoside diphosphate kinase B, UV excision repair protein, insulin like growth factor receptor II, transcription initiation factor TFIID  [ **TBP** ]  subunit, growth factor receptor bound protein 2, UV excision repair protein, glutathione peroxidase, Y box binding protein 1 and heat shock protein 86.  Ref: 10946852 Life Sci, 2000
- c  **fos**  and c Ha ras gene expression was enhanced in liver, but not kidney, slices by BaP  [ **TBP** ] . c jun mRNA levels were decreased in liver and kidney slices, although the effect was earlier 0.5 h in liver slices compared to kidney slices.  Ref: 9852999 Toxicol Appl Pharmacol, 1998
- The association between Tat,  **Fos** , and Jun would position Tat proximal to the viral TATA box, where the visna virus Tat activation domain could contact  **TBP**  to activate viral transcription.  Ref: 9847304 J Virol, 1999
- It is the regulatory subunit of the DNA dependent protein kinase that phosphorylates many proteins, including SV 40 large T antigen, p53, RNA polymerase II, RP A, topoisomerases, hsp90, and many transcription factors such as c Jun, c  **Fos** , oct 1, sp 1, c Myc, TFIID  [ **TBP** ] , and many more.  Ref: 10755664 Crit Rev Biochem Mol Biol, 2000
